# Sarcopenic obesity definitions and their associations with physical frailty in older Brazilian adults: data from the SARCOS study

**DOI:** 10.20945/2359-3997000000587

**Published:** 2023-01-31

**Authors:** Alberto Frisoli, Gustavo Duque, Angela T Paes, Amanda Rocha Diniz, Eliene Lima, Elaine Azevedo, Valdir Ambrósio Moises

**Affiliations:** 1 Universidade Federal de São Paulo Escola Paulista de Medicina Divisão de Cardiologia São Paulo SP Brasil Ambulatório de Cardiologia Geriátrica, Divisão de Cardiologia, Escola Paulista de Medicina, Universidade Federal de São Paulo, São Paulo, SP, Brasil.; 2 Universidade Federal de São Paulo Escola Paulista de Medicina Divisão de Cardiologia São Paulo SP Brasil Grupo de Pesquisa em Vulnerabilidade a Doenças do Idoso – Divisão de Cardiologia, Escola Paulista de Medicina, Universidade Federal de São Paulo, São Paulo, SP, Brasil.; 3 Universidade Federal de São Paulo Faculdade de Medicina Divisão de Cardiologia São Paulo SP Brasil Divisão de Cardiologia, Faculdade de Medicina, Universidade Federal de São Paulo, São Paulo, SP, Brasil.; 4 University of Melbourne Melbourne Medical School Australian Institute for Musculoskeletal Science Victoria Australia Australian Institute for Musculoskeletal Science, Melbourne Medical School, University of Melbourne, Victoria, Australia.; 5 Universidade Federal de São Paulo Departamento de Estatística São Paulo SP Brasil Departamento de Estatística, Universidade Federal de São Paulo, São Paulo, SP, Brasil.

**Keywords:** Sarcopenia, frailty, obesity, elderly

## Abstract

**Objective:**

To identify the obesity diagnosis with the highest association with physical frailty associated with sarcopenia EWGSOP II (sarcopenic obesity).

**Subjects and methods:**

We performed a cross-sectional analysis of 371 community-dwelling older adults. Appendicular skeletal lean mass and total body fat (TBF) were assessed using dual-energy x-ray absorptiometry, and physical frailty was defined using Fried's criteria. The phenotypes were identified according to the presence of sarcopenia by EWGSOP II and obesity, which was diagnosed using two concepts: BMI obesity (BMI ≥ 30 kg/m^2^) and TBF obesity (percentage of TBF ≥ 35% for women and ≥ 25% for men). Finally, the association of each group with physical frailty was evaluated.

**Results:**

The mean age was 78.15 ± 7.22 years. Sarcopenia EWGSOP II was diagnosed in 19.8% (n = 73), body mass index obesity was identified in 21.8% (n = 81), TBF obesity was identified in 67.7% (n = 251), and physical frailty was identified in 38.5% (n = 142). In a regression analysis for frailty, sarcopenic TBF obesity presented an odds ratio of 6.88 (95% confidence interval 2.60-18.24; p < 0.001).

**Conclusion:**

In older Brazilian adults, sarcopenic obesity diagnosed by TBF obesity has a robust association with frailty and is independent of body mass index.

## INTRODUCTION

In 2001, Fried and cols. hypothesized that sarcopenia (i.e., reduced muscle mass) would be a primary factor in the pathophysiology of the frailty syndrome (1). Studies later showed that the relationship between frailty and sarcopenia varies significantly according to gender, presence of comorbidities, and functional status (2–5). With the emergence of the new definitions of sarcopenia, including impaired physical performance (weakness or low gait speed) (2,6), a more significant interaction between sarcopenia and frailty has been observed, suggesting that reduced muscle mass does not have the anticipated influence on the etiology of frailty and is only significant when interacting with other factors or clinical conditions.

High body mass index (BMI) is associated with lower mobility, reduced physical activity, and consequently, less energy expenditure and loss of muscle mass (4,5,7). Furthermore, excess abdominal fat has been associated with higher levels of markers of oxidative stress and inflammatory cytokines, independent of BMI (8), with a consequent decrease in muscle function. Obesity (via mechanical and physiological factors) appears to act synergistically with sarcopenia in relation to frailty (5). Moreover, the association of obesity and sarcopenia (i.e., sarcopenic obesity) has been associated with impaired physical capacity and reduced muscle strength (8,9), functional loss, and a higher incidence of frailty (9,10). However, in clinical practice, obesity (in terms of BMI or otherwise) is not generally considered in the diagnosis of sarcopenia or frailty status. This omission introduced bias regarding the epidemiological significance of the association of obesity with sarcopenia. In addition, there are several criteria for sarcopenia and obesity, and it is challenging to identify the sarcopenic obesity phenotype with the most significant association with physical frailty in older individuals (11,12).

To solve this challenge, we hypothesized that sarcopenia [diagnosed according to the revised European Working Group on Sarcopenia in Older People – EWGSOP II (13)] would be more frequently associated with physical frailty when interacting with obesity than with sarcopenia or obesity alone. To test this hypothesis, we evaluated the association between the phenotypes derived from the interaction between sarcopenia EWGSOP II and two obesity diagnoses (i.e., high BMI and a high percentage of total body fat – TBF) with physical frailty.

## SUBJECTS AND METHODS

### Study design

This was a cross-sectional analysis of the SARCopenia and OSteoporosis in older adults with cardiovascular diseases study (SARCOS study). This one-year prospective cohort study investigated the association of sarcopenia and osteoporosis as a common pathway to disability and physical frailty in older adults in an outpatient community-dwelling setting (14).

### Sample

Our sample consisted of older adults from a geriatric cardiology outpatient clinic. Inclusion criteria were age over 60 years and one or more cardiovascular diseases. Both genders and all ethnic groups were considered.

**Figure 1:**
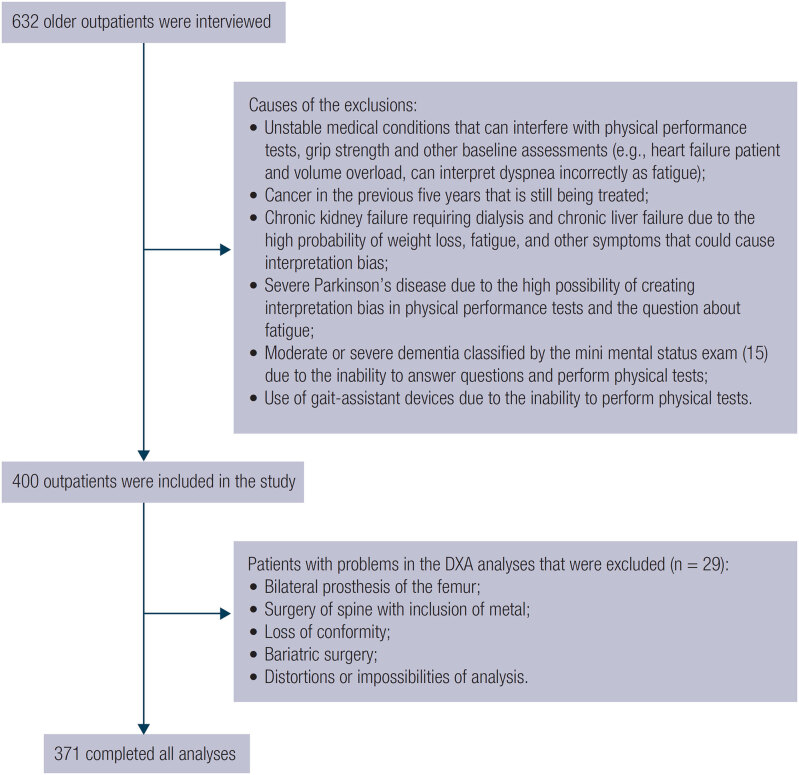
Sample selection flowchart

The exclusion criteria and the sample selection flowchart are shown in Figure 1.

After signing informed consent, patients underwent a physical exam, physical performance tests, and dual- energy X-ray absorptiometry (DXA) (GE Lunar; DPX- MD 73477, GE Medical Systems, Madison, WI, USA).

The Ethics Committee at our institution approved the study (approval nº 682659), and all participants provided informed consent.

### Variables recorded at baseline

#### The following disease data were obtained from medical records

##### Medical history

- Cardiovascular diseases: arterial hypertension, diabetes mellitus, previous myocardial infarction (over six months before the study), angina, heart failure, previous stroke (over six months before the study), peripheral arterial disease.
- Chronic diseases: osteoarthritis, non-dialytic chronic kidney disease, chronic obstructive pulmonary disease, and history of minimal trauma fracture over the previous ten years.

##### Lifestyle factors and other information

We recorded demographic data, medications (per day), self-reported current or past smoking and pack-years (where applicable), and self-reported current or past alcohol consumption.

#### Body composition measures

All subjects underwent DXA to measure total body composition differentiated by the software in terms of total and regional distribution (left arm and leg; right arm and leg; trunk), lean mass and fat mass (in kg), and percentage. Appendicular skeletal lean mass (ASLM) was obtained by the sum of arms and legs lean mass (kg) divided by height squared (m^2^) (13).

The percentage of TBF was determined by the sum of the fat percentage of arms, legs, trunk, and pelvis in relation to total body mass.

BMI was calculated with weight (kg) divided by height squared (m^2^) (16).

In our laboratory, the *in vivo* precision (coefficient of variation [CV%]) based on three repeated scans of 15 women and 15 men with repositioning was 1.62% for TBF percentage and 1.64% for ASLM (17,18).

#### Muscle strength

Muscle strength was evaluated as the grip strength of the dominant hand, measured using a hand-held dynamometer (Jamar; TEC, Clifton, NJ, USA) determined by three measurements; only the maximum values were reported. The measurement was made with the patient sitting with the elbow supported and the shoulder relaxed with the forearm at 90 degrees to the arm (19).

#### Diagnosis of sarcopenia

Sarcopenia was diagnosed according to EWGSOP II (13), where subjects with handgrip strength equivalent to or lower than 27 kg for men and 16 kg for women and ASLM < 7.0 kg/m^2 ^for men and < 6.0 kg/m^2^ for women were considered sarcopenic.

#### Diagnosis of obesity

Obesity was assessed and diagnosed using the following concepts:

- BMI obesity: BMI ≥ 30 kg/m^2^ as recommended by the World Health Organization (16).
- TBF obesity: percentage of TBF ≥ 35% for women and ≥ 25% for men (20).

#### Phenotype definitions of the sarcopenic obesity

The phenotypes of sarcopenic obesity were developed according to the presence or absence of both sarcopenia and TBF (21) or BMI obesity, as characterized below.

##### Sarcopenia and BMI obesity groups

1. Sarcopenic BMI obesity: subjects with sarcopenia EWGSOP II and BMI obesity.
2. Sarcopenia without BMI obesity: subjects with sarcopenia EWGSOP II and without BMI obesity.
3. BMI obesity: subjects with BMI obesity and without sarcopenia EWGSOP II.
4. BMI controls: subjects without sarcopenia EWGSOP II or BMI obesity.

##### Sarcopenia and TBF obesity group

1. Sarcopenic TBF obesity: subjects with sarcopenia EWGSOP II and TBF obesity.
2. Sarcopenia without TBF obesity: subjects with sarcopenia EWGSOP II and without TBF obesity.
3. TBF obesity: subjects with TBF obesity and without sarcopenia EWGSOP II.
4. TBF Controls: subjects without sarcopenia EWGSOP II or TBF obesity.

#### Frailty

Frailty was defined according to Fried's criteria (1): shrinking (unintentional loss of ≥ 10 pounds in the previous year or [at follow-up] a loss of ≥ 5% of body weight in the prior year), weakness (hand grip strength in the lowest 20% at baseline, adjusted for gender; i.e., hand grip strength ≤ 24 kg for men and ≤ 14 kg for women) (14), “adapted” low physical activity (i.e., the inability to complete the five-repetition sit-to-stand in the chair stand test), slowness (walking speed ≤ 0.8 m/s), and exhaustion (affirmative response to the question “Do you feel fatigued most of the time?”).

Individuals with three or more of the five criteria were considered frail; one or two were considered pre- frailty; patients with no criteria were considered robust. In the analyses, we categorized frailty phenotypes into frail and non-frail patients, i.e., pre-frail patients were grouped with robust patients.

### Statistical analysis

Categorical data were expressed as frequencies and percentages and numerical variables as means ± standard deviations. The Chi-square test and analysis of variance were used to compare the phenotype groups (qualitative and quantitative variables, respectively). The Levene test was used to check the homogeneity of variances assumption. The normality of distribution was checked using descriptive statistics, normality plots, and the Kolmogorov-Smirnov test.

Logistic regression was used to evaluate the proposed phenotypes’ association with physical frailty. Models were fitted separately for each obesity criterion (BMI and TBF obesity) due to the overlap between groups. We used the control group of each sarcopenia and obesity phenotypes as the reference categories. Results from logistic models were presented as odds ratios (ORs) and 95% confidence intervals (CIs). The ORs were adjusted for statistically significant frailty-associated variables (age, female gender, heart failure, and previous falls) and agreed with previously described variables (22,23). The Hosmer-Lemeshow test was performed to assess the goodness of fit for all the logistic regression models. SPSS version 22.0 (SPSS, Inc., Chicago, IL, USA) statistical software package was used for all analyses. Differences with P < 0.05 were considered significant.

## RESULTS

The average age of our sample was 78.15 (±7.22) years, 53.2% (n = 189) women, 66.6% (n = 293) Caucasian, and 30.5% (n = 134) Black. Physical frailty was diagnosed in 38.5% (n = 143) and prefrailty in 51.5% (n = 190); 10% (n = 37) were robust and 61.5% (n = 228) were non-frail.

Sarcopenia was diagnosed in 19.8% (n = 73) participants. The prevalence of obesity by BMI was 21.8% (n = 81), while obesity by TBF was three times more prevalent at 67.7% (n = 251). Almost 22% (n = 80) of the subjects presented both diagnoses of obesity, and only one subject with obesity by BMI did not have obesity by TBF.

### Sarcopenic obesity, sarcopenia without obesity, and obesity phenotypes

There was significant variability in the prevalence of sarcopenic obesity and sarcopenia without obesity phenotypes according to the obesity diagnostic criteria. Among individuals with sarcopenia, 72.6% (n = 53) had TBF obesity, and only 8.2% (n = 6) had BMI obesity. In individuals with sarcopenia without obesity, 91.8% (n = 67) of individuals had no BMI obesity and 27.4% (n = 20) TBF obesity.

Interestingly, 70.15% (n = 47) of individuals with sarcopenia without BMI obesity were also classified as sarcopenic TBF obesity, demonstrating substantial overlap with the obesity criteria. The characteristics of each phenotype are displayed in Tables 1 and 2.

**Table 1:** Demographic characteristics of subjects from the association between BMI obesity and sarcopenia

BMI Obesity							
Variables		Total, %(n)	Sarcopenic obesity,1.6% (n = 6)	Sarcopenia without obesity, 18.1% (n = 67)	Obesity only, 21.8% (n = 81)	Controls, 58.5% (n = 217)	p-value*
Gender							
	Men	46.1(171)	16.7(1)	49.3 (33)	38.3 (31)	48.8 (106)	0.174
	Women	53.9 (200)	83.3 (5)	50.7 (34)	61.7 (50)	51.2 (111)	
Mean age (years) ± SD		78.15 ± 7.22	81.25 ± 6.84	82.65 ± 6.70	75.82 ± 6.99	77.79 ± 6.94	<0.001
Race							
	Caucasian	66.7 (251)	83.3 (5)	71.6 (48)	66.7 (54)	66.4 (144)	**0.040**
	Black	29.1 (108)	16.7 (1)	17.9 (12)	33.3 (27)	31.3 (68)	
	Asian	3.2 (12)	0 (0.0)	10.4 (7)	0.0 (0)	2.3 (5)	
Diabetes mellitus		40.2 (149)	50.0 (3)	37.3 (25)	51.6 (41)	36.9 (80)	0.162
Hypertension		93.3 (349)	100.0 (6)	94.0 (63)	95.1 (77)	92.2 (200)	0.729
Heart failure		28.3 (105)	50.0 (3)	31.3 (21)	33.3 (27)	24.9 (54)	0.267
Chronic kidney disease		17.8 (66)	20.0 (1)	20.9 (14)	16.0 (13)	17.5 (38)	0.893
Prior cancer		13.5 (50)	16.7 (1)	20.9 (14)	9.9 (8)	12.4 (27)	0.210
Stroke		10.8 (40)	33.3 (2)	11.9 (8)	8.6 (7)	10.6 (23)	0.283
Osteoarthritis		35.1 (129)	50.0 (3)	29.9 (20)	46.3 (37)	32.2 (69)	0.087
Falls in prior six months		28.8 (107)	16.7 (1)	37.3 (25)	25.9 (21)	27.6 (60)	0.356
Alcohol history,		13.2 (49)	16.7 (1)	11.9 (8)	9.9 (8)	14.7 (32)	0.725
Smoking history		47.3 (175)	40.0 (2)	44.8 (30)	43.2 (35)	49.8 (108)	0.730
Physical performance							
	Grip strength (kg mean ± SD)	23.64 ± 7.87	15.25 ± 3.01	16.68 ± 5.24	24.35 ± 7.49	25.38 ± 7.57	**<0.001**
	Walking speed (m/s mean ± SD)	0.78 ± 0.38	0.42 ± 0.15	0.61 ± 0.29	0.76 ± 0.30	0.83 ± 0.41	**<0.001**
Body composition							
	Total body fat % mean ± SD	38.78 ± 9.55	49.10 ± 5.85	36.28 ± 9.62	45.87 ± 6.78	36.57 ± 9.01	**<0.001**
	ASLM (kg/m^2^ mean ± SD)	6.66 ± 1.16	5.91 ± 0.37	5.64 ± 0.73	7.52 ± 1.10	6.69 ± 1.03	**<0.001**
	BMI (kg/m^2^ mean ± SD)	27.03 ± 4.66	32.02 ± 1.56	23.19 ± 3.30	33.26 ± 2.99	25.55 ± 2.93	**<0.001**

ASLM: appendicular skeletal lean mass index; BMI: body mass index; TBF: total body fat percentage.

* The Pearson's Chi-square test was used for categorical data and one-way analysis of variance for continuous variables.

**Table 2:** Demographic characteristics of subjects from the association between total body fat obesity and sarcopenia

Total body fat Obesity							
Variables		Total, % (n = 371)	Sarcopenic obesity,14.1% (n = 53)	Sarcopenia without obesity, 5.4% (n = 20)	Obesity only, 67.7% (n = 251)	Controls, 12.7% (n = 47)	p-value*
Gender							
	Men	46.1 (171)	54.7(29)	25.0(5)	46.2(116)	44.7(21)	0.155
	Women	53.9 (200)	45.3 (24)	75.0 (15)	53.8 (135)	55.3 (26)	**0.159**
Mean age (years) mean ± SD		78.15 (±7.22)	83.06 (±6.84)	80.26 (±5.58)	77.09 (±7.00)	79.45 (±6.10)	**<0.001**
Race							
	Caucasian	66.7 (251)	75.5(40)	65.0 (13)	69.7(175)	48.9 (23)	**0.001**
	Black	29.1 (108)	17.0 (9)	20.0 (4)	29.1 (73)	46.8 (22)	
	Asian	3.2 (12)	7.5 (4)	15.0 (3)	1.2 (3)	4.3 (47)	
Diabetes mellitus		40.2 (149)	41.5 (22)	30.0 (6)	42.6 (107)	29.8 (107)	0.303
Hypertension		93.3 (349)	92.5 (49)	100.0 (20)	93.2 (254)	91.5 (43)	0.673
Heart failure		28.3 (105)	32.1 (17)	35.0 (7)	27.9 (70)	23.4 (11)	0.715
Chronic kidney disease		17.8 (66)	23.1 (12)	15.0 (3)	17.1 (43)	17.0 (8)	0.777
Prior cancer		13.5 (50)	26.4 (14)	5.0 (1)	11.2 (28)	14.9 (7)	0.018
Stroke		10.8 (40)	13.2 (7)	15.0 (3)	10.0 (25)	10.6 (5)	0.847
Osteoarthritis		35.1 (129)	32.1 (17)	30.0 (6)	38.2 (95)	24.4 (11)	0.303
Falls in prior six months		28.8 (107)	32.1 (17)	45.0 (9)	27.1 (68)	27.7 (13)	0.361
Alcohol history		13.2 (49)	15.1 (8)	5.0 (1)	12.7 (32)	17.0 (8)	0.571
Smoking history		47.3 (175)	46.2 (24)	40.0 (8)	46.2 (116)	57.4 (27)	0.479
Physical performance							
	Grip strength (kg mean ± SD)	23.64 (±7.87)	16.36 (±5.08)	17.47 (±5.42)	24.55 (±7.59)	25.57 (±7.08)	**<0.001**
	Walking speed (m/s mean ± SD)	0.78 (±0.38)	0.56 (±0.25)	0.71 (±0.38)	0.79 (±0.40)	0.91 (±0.36)	**<0.001**
Body composition							
	Total body fat % mean ± SD	38.78 (±9.55)	40.48 (±7.67)	23.13 (±6.17)	40.98 (±7.60)	20.86 (±5.30)	**<0.001**
	ASLM (kg/m^2^ mean ± SD)	6.66 (±1.16)	5.67 (±0.74)	5.63 (±0.58)	6.92 (±1.12)	6.88 (±0.99)	**<0.001**
	BMI (kg/m^2^ mean ± SD)	27.03 (±4.66)	24.87 (±3.66)	19.59 (±2.07)	28.27 (±4.22)	21.69 (±2.45)	**<0.001**

ASLM: appendicular skeletal lean mass index; BMI: body mass index; TBF: total body fat percentage.

* The Pearson's Chi-square test was used for categorical data and one-way analysis of variance for continuous variables.

### Sarcopenic obesity

The individuals with sarcopenic BMI obesity (Table 1) were predominantly women, Caucasian, and very old (81.25 ± 6.84 years). Half had diabetes, heart failure, and osteoarthritis, and these patients had a higher prevalence of hypertension and stroke history.

In the sarcopenic TBF obesity phenotype (Table 2), both genders had similar prevalence, and the mean age was similar to the sarcopenic BMI obesity group. These patients had more previous cancers and two-fold more falls; however, mean grip strength and walking speed were higher than the sarcopenic BMI obesity group.

Regarding body composition, the sarcopenic BMI obesity presented a higher BMI and TBF percentage but a similar appendicular muscle mass index compared to the sarcopenic TBF obesity. All subjects with sarcopenic BMI obesity had TBF obesity.

### Sarcopenia without obesity

The phenotypes of the subjects with sarcopenia without obesity were not characterized by a specific gender, age, or race. Previous cancer was almost two-fold more common in individuals with sarcopenia without BMI obesity, while those with sarcopenia without TBF obesity had a higher prevalence of previous stroke and a lower prevalence of smoking. TBF percentage was significantly higher among those with sarcopenia without BMI obesity than sarcopenia without TBF obesity, while the amount of appendicular muscle mass was similar in both phenotypes. Among the phenotypes of sarcopenia without obesity, all subjects with sarcopenia without TBF obesity were also diagnosed with sarcopenia without BMI obesity.

#### Physical frailty

The demographic characteristics, comorbidities, physical performance, and body composition of frailty and non-frailty subjects are displayed in Table 3. In frail subjects, sarcopenic TBF obesity occurred in 49.7% (n = 71), and sarcopenic BMI obesity occurred in 14.7% (n = 21). In non-frail subjects, the tendency remained the same, with ten times more individuals with sarcopenic TBF obesity than sarcopenic BMI obesity (Table 4). Sarcopenia without obesity phenotypes showed an opposite trend. Sarcopenia BMI obesity was three times more frequent than sarcopenia TBF obesity in the elderly with frailty (36.4% *vs.* 9.8%).

**Table 3:** Demographic characteristics of frail and non-frail subjects

		Total, % (n = 371)	Frailty, % (n = 143)	Non frailty % (n = 228)	p*
Gender					
	Men	46.6 (173)	39.9 (57)	50.9 (116)	0.025
	Women	53.4 (198)	60.1 (80)	49.1 (112)	
Age, Years		78.2 (6.9)	81.1(6.7)	76.3 (6.4)	<0.001
Race					
	Caucasian	68.2 (253)	70.6 (101)	66.7 (152)	0.671
	Black	28.6 (106)	25.9 (37)	30.3 (69)	
	Asian	3.2 (12)	3.5 (5)	3.1 (7)	
Hypertension		93.3 (346)	93.7 (134)	93 (212)	0.835
Diabetes mellitus		40.7 (151)	39.9 (57)	41.2 (94)	0.829
Heart failure		28.3 (105)	35.0 (50)	24.1 (55)	0.025
Osteoarthritis		35.7 (131)	37.3 (53)	34.7 (78)	0.655
Falls in the prior six months		28.3 (105)	34.3 (49)	24.6 (56)	0.645
Stroke		10.8 (40)	11.9 (17)	10.1 (23)	0.609
Chronic kidney diseases		18.1 (67)	21.1 (30)	16.2 (37)	0.267
Previous cancer		13.2 (49)	14.0 (20)	12.7 (29)	0.759
Alcohol history		13.2 (49)	12.6 (18)	13.6 (31)	0.875
Smoke history		47.3 (175)	41.5 (59)	50.9 (116)	0.087
Physical performance					
	Grip strength (kg mean ± SD)	23.3 (7.8)	18.6 (7.0)	26.3 (6.8)	<0.001
	Walking speed (m/s mean ± SD	0.76 (0.4)	0.53 (0.2)	0.91 (0.4)	<0.001
Body composition					
	Total body fat % mean ± SD	38.23 (9.4)	38.95 (8.7)	37.77 (9.8)	0.235
	ASLM (kg/m^2^ mean ± SD)	0.42 (0.5)	0.50 (0.5)	0.36 (0.5)	0.009
	BMI (kg/m^2^ mean ± SD)	26.7 (4.6)	26.19 (4.6)	27.16 (4.5)	0.053

* The Pearson's Chi-square test was used for categorical data and one-way analysis of variance for continuous variables.

**Table 4:** Prevalence of sarcopenic (BMI/TBF) obesity, sarcopenia without (BMI/TBF) obesity, (BMI/TBF) obesity only, and controls phenotypes in frail and non-frailty older adults

		Total % (n = 371)	Frailty 38.5% (n = 143)	Non-frailty 61.5% (n = 228)	p*
Sarcopenia and BMI obesity phenotypes					
	Sarcopenic BMI obesity	1.6 (6)	3.5 (5)	0.4 (1)	<0.001
	Sarcopenia without BMI obesity	18.1 (67)	36.4 (52)	6.6 (15)	
	BMI obesity only	21.8 (81)	14.7 (21)	26.3 (21)	
	BMI controls	58.5 (217)	45.5 (65)	66.2 (152)	
Sarcopenia and TBF obesity phenotypes					
	Sarcopenic TBF obesity	14.3 (53)	30.1 (43)	4.4 (10)	<0.001
	Sarcopenia without TBF obesity	5.4 (20)	9.8 (14)	2.6 (6)	
	TBF obesity only	67.7 (251)	49.7 (71)	78.9 (180)	
	TBF controls	42.7 (47)	10.5 (15)	14.0 (32)	

* Pearson's Chi-square.

#### Logistic regression analysis

In the adjusted logistic regression analyses for physical frailty, sarcopenic TBF obesity showed a robust and significant association that did not change after adjusting for confounding variables, while sarcopenic BMI obesity did not maintain statistical significance after adjustments (Table 5). The two phenotypes of sarcopenia without obesity had a significant association with frailty; however, sarcopenia without BMI obesity showed a higher value and a narrower confidence interval after adjustments than sarcopenia without TBF obesity. The phenotypes of obesity alone showed a tendency toward a negative association with frailty, but none reached statistical significance.

**Table 5:** Unadjusted and adjusted regression analyses for frailty, from sarcopenic (BMI/TBF) obesity, sarcopenia without (BMI/TBF) obesity, (BMI/TBF) obesity only phenotypes

		Odds ratio (unadjusted)	Odds ratio (adjusted)
Sarcopenia and BMI obesity			
	Sarcopenic BMI obesity	11.69 (1.34-102.05; p = 0.026)	8.39 (0.89-78.51; p = 0.062)*
	Sarcopenia without BMI obesity	8.10 (4.25-15.43; p < 0.001)	6.43 (3.26-12.67; p < 0.001)*
	BMI obesity only	0.81 (0.46-1.45; p = 0.495)	0.95 (0.51-1.76; p = 0.879)
	Controls (Reference)	1	1
Sarcopenia and TBF obesity			
	Sarcopenic TBF obesity	9.17 (3.69-23.05; p < 0.001)	7.25 (2.74-19.17; p < 0.001)**
	Sarcopenia without TBF obesity	4.97 (1.59-15.50; p = 0.006)	3.35 (1.01-11.11; p = 0.047)**
	TBF obesity only	0.84 (0.43-1.64; p = 0.615)	0.83 (0.41-1.69; p = 0.620)**
	Controls (Reference)	1	1

Dependent variable: frailty

* Adjusted for age, heart failure, previous falls, and female gender.

** Adjusted for the same variables in the footnote* plus BMI.

## DISCUSSION

To our knowledge, this study is the first to evaluate the association of the frailty phenotype with sarcopenic obesity diagnosed according to EWGSOP II.

The main contribution of this study was the finding that the excess percentage of body fat has a strong synergistic action with sarcopenia concerning frailty, independent of BMI. When sarcopenia is assessed without TBF obesity, there was a positive relationship between sarcopenia and frailty (although less than initially expected), suggesting that we might overestimate the actual relationship between sarcopenia and frailty and possibly with other vulnerability syndromes. In our population, the number of sarcopenic TBF obesity participants was almost three times higher than subjects with sarcopenia without TBF obesity, suggesting that a large proportion of the elderly diagnosed as sarcopenic in fact have sarcopenic TBF obesity, and this fact could trigger misinterpretations of epidemiological data related to sarcopenia. Moreover, it is critical to highlight that because the percentage of TBF is not routinely assessed in DXA exams, these patients might be underdiagnosed in clinical practice. One of the physiological reasons for the functional deterioration of the skeletal muscle system in the sarcopenic TBF obesity phenotype is the pro-inflammatory action of excess body fat. The presence of a large amount of fat (i.e., total body, trunk, or visceral) promotes greater production of inflammatory cytokines of the immune system (interleukin-6, tumor necrosis factor-alpha, c-reactive protein) (24–26) that act as muscle growth inhibitors promoting the loss of muscle mass and function (27).

The TBF obesity phenotype did not show a significant association with frailty; on the contrary, it had a protective tendency (Table 5). This finding might be due to the absence of low skeletal muscle mass and weakness, which strongly correlate with the frailty criteria such as low gait speed, low energy expenditure, and muscle weakness.

Data on the association between a high percentage of body fat with sarcopenia and its relationship with vulnerability outcomes are controversial, mainly due to the variation of the diagnostic criteria of sarcopenia, regardless of whether physical performance tests are involved (28–30). When defining sarcopenia by the criterion of low muscle mass and obesity by body fat percentage higher than 40%, Rolland and cols. observed that women with sarcopenic obesity were 2.54 times more likely to report mobility difficulties than those without these diagnoses; furthermore, sarcopenia without obesity was not associated with mobility impairments (4). In another study with sarcopenia defined by low muscle mass and obesity by fat mass higher than 30%, Hirani and cols. observed that sarcopenic obesity and sarcopenia without obesity had similar values of risk for incidental frailty [OR = 2.12 (CI: 1.42-3.18, p < 0.0001) and OR = 2.00 (CI: 1.42-2.82, p < 0.0001), respectively]; this was also true for disabled activities of daily living [OR = 1.30 (CI: 0.84-1.99; p = 0.24) and OR = 1.58 (CI: 1.12, 2-24; p = 0.01), respectively] and disabled instrumental activities of daily living [OR = 1.36 (CI 1.05-1.76; p = 0.02) and 1.32 (CI: 1.06-1.64; p = 0.01), respectively] (11). By contrast, Aggio and cols. showed that sarcopenic obesity diagnosed using the original concept of EWGSOP and obesity diagnosed using waist circumference > 102 cm was not associated with low physical performance in a population of 7,735 men with an average age of 77 years (30).

We observed a high proportion of subjects with frailty and few robust subjects. There are several possible explanations for these results. Our sample was composed of subjects attending a cardiovascular disease outpatient clinic at a tertiary center, a service that does not serve low-complexity patients and primarily cares for those with high rates of comorbidities and their consequences. Another reason might be the low average grip strength and walking speed, which increases the percentage of weak patients and those with lower walking speed. Another factor is the sense of fatigue/exhaustion (1), which is also high due to the high prevalence of heart failure with preserved or reduced ejection fraction. Other factors, such as polypharmacy, low income, and poor education, might also be associated with frailty (1).

Our population had a high prevalence of cardiovascular disease (hypertension, diabetes, and heart failure), which strongly correlates with obesity (31,32). In addition, visceral or TBF is a risk factor for various types of cancer, diabetes, and mortality (33–35), and the presence of these diseases significantly increases the risk of frailty. Sarcopenic and obese individuals are at higher risk for cardiovascular diseases; however, when both are present, the risk appears to be even more significant, even though longitudinal studies have shown conflicting results depending on the obesity criterion used (35,36).

BMI obesity did not appear to have the same synergistic effect with sarcopenia as TBF obesity concerning frailty; however, we believe that this phenomenon is due to the lower prevalence of obesity by BMI than TBF obesity (1:3) and, consequently, the reduced number of older adults with sarcopenic BMI obesity (n = 6). Furthermore, the sarcopenia without BMI obesity phenotype showed an association with frailty like that of sarcopenic TBF obesity [6.50 (3.31-12.76; p < 0.001) vs. 6.88 (2.60-18.24; p < 0.001), respectively]. Nevertheless, we must exercise caution when interpreting these results, as 70.15% (n = 47) of the elderly with sarcopenia without BMI obesity phenotype had TBF obesity; that is, they simultaneously had sarcopenic TBF obesity. The lack of association between sarcopenic BMI obesity and syndromes of clinical vulnerability has been previously reported (33,35); as in our cohort, there was a small sample of elderly individuals with a BMI above 30 kg/m^2^.

Several studies identified BMI obesity as a risk factor for the incidence of functional loss and mortality (37–40). Our results corroborate that obesity in isolation (without sarcopenia) does not appear to contribute to a phenotype of physical vulnerability (29,30,41). This finding highlights the fact that, in older adults, being obese alone without sarcopenia may not result in clinical deterioration.

Among the study limitations are the relatively small sample size and the elevated mean age (mean age of 78 years), which does not allow the generalization of our findings to a younger population. We did not perform Time Up and go Test and Short Physical Performance Battery in our population; consequently, we were limited in diagnosing sarcopenia and further analyzing the frailty phenotype. Also, our study population was derived from a cardiovascular outpatient clinic; therefore, our findings might not be generalized to the general population. Finally, this study was a cross- sectional analysis and, as such, does not allow the establishment of cause-and-effect relationships between the presence of sarcopenia, obesity, and frailty.

In summary, we found that sarcopenia comprises two phenotypes with different morphological and functional characteristics (sarcopenia with and without obesity). The obesity criterion (BMI or TBF) significantly influences the prevalence and association with clinical vulnerability syndromes, and the presence of TBF obesity is more critical in determining the association with frailty in older adults with sarcopenia.

These findings highlight the importance of establishing a standard criterion for diagnosing sarcopenic obesity and the need for systematic differentiation between sarcopenia with and without obesity in clinical practice. The measurement of muscle mass and percentage of TBF can be performed concurrently with screening tests for osteoporosis, while the assessment of muscle strength can be easily performed in the medical office.

In conclusion, TBF obesity in Brazilian older adult outpatients with sarcopenia is associated with physical frailty, regardless of BMI. BMI obesity in patients with sarcopenia does not appear to be sufficient to establish a significant association with frailty.
